# A gut aging clock using microbiome multi-view profiles is associated with health and frail risk

**DOI:** 10.1080/19490976.2023.2297852

**Published:** 2024-01-30

**Authors:** Hongchao Wang, Yutao Chen, Ling Feng, Shourong Lu, Jinlin Zhu, Jianxin Zhao, Hao Zhang, Wei Chen, Wenwei Lu

**Affiliations:** a State Key Laboratory of Food Science and Resources, Jiangnan University, Wuxi, Jiangsu, China; b School of Food Science and Technology, Jiangnan University, Wuxi, Jiangsu, China; c The Affiliated Wuxi People’s Hospital of Nanjing Medical University, Wuxi People’s Hospital, Wuxi Medical Center, Nanjing Medical University, Wuxi, Jiangsu, China; d Wuxi Translational Medicine Research Center and Jiangsu Translational Medicine Research Institute Wuxi Branch, Wuxi, Jiangsu, China; e National Engineering Research Center for Functional Food, Jiangnan University, Wuxi, Jiangsu, China

**Keywords:** Gut microbiome, metagenomics, machine learning, aging, frailty

## Abstract

Age-related changes in the microbiome have been reported in previous studies; however, direct evidence for their association with frailty is lacking. Here, we introduce biological age based on gut microbiota (gAge), an integrated prediction model that integrates gut microbiota data from different perspectives with potential background factors for aging assessment. Simulation results show that, compared with a single model, the ensemble model can not only significantly improve the prediction accuracy, but also make full use of the data in unpaired samples. From this, we identified markers associated with age development and grouped markers into accelerated aging and mitigated aging according to their effect on the prediction. Importantly, the application of gAge to an elderly cohort with different frailty levels confirmed that gAge and its predictive residuals are closely related to the individual’s health status and frailty stage, and age-related markers overlap significantly with disease and frailty characteristics. Furthermore, we applied the gAge prediction model to another independent cohort of the elderly population for aging assessment and found that gAge could effectively represent the aging population. Overall, our study explains the association between the gut microbiota and frailty, providing potential targets for the development of gut microbiota-based targeted intervention strategies for aging.

## Introduction

Aging is a continuous and gradual process over a long time scale in humans. With time, the organism continuously exhibits an overall decline in all aspects of function, including changes in molecular, biochemical, and physiological processes; organ function; and disease susceptibility, which ultimately lead to death. Although it is very critical and has been extensively analyzed, a comprehensive view of the changes and mechanisms that affect human lifespan is lacking. This is because human health and longevity are affected by genetic, epigenetic, and environmental factors and the complex interactions among these factors.^1^ In this regard, the microbiome is considered a potentially key player that is closely related to human health and the aging process.

Over the past several years, there has been a research shift in the understanding of the gut microbiota, which is an important factor in maintaining human health and has been associated with many diseases. In addition, the gut microbiome has time-related trajectories of structural and functional changes similar to those of the host organism, and these changes with age may predispose individuals to aging-related diseases such as osteoporosis, parkinsonism.^2,3^ A typical situation is the imbalance in microbial homeostasis, which alters gut permeability and immune function and may lead to inflammation. Chronic inflammation is the most common biological characteristic of aging and age-related disease.^4,5^ In other cases, it may also increase the risk of metabolic diseases, neurological disorders, and cancer. More importantly, microbes are not only affect but also responsive the host status.^6^ One well-known example is short-chain fatty acids (SCFA), a series of human gut microbial metabolites that help regulate appetite and anti-inflammatory effects.^7^ This reciprocal relationship makes it valuable to examine the association between gut microbiota and the aging process of the host. Revealing the interaction between gut microbiota and age can help identify microorganisms that are potentially associated with host aging and contribute to the development of targeted therapeutic strategies for aging interventions.^8,9^

Although numerous studies have shown that certain microbial distributions are age-specific or associated with aging, research in the field of gut aging has produced relatively limited results.^10^ The main problems that have caused the current dilemma are the complexity of the gut microbiome, lack of a comprehensive understanding of the microbes, including their diverse composition and structures, and the influence of exogenous factors. However, it is possible to obtain more systematic information regarding the complex gut microecosystem with the help of new analytical tools such as whole genomic sequencing and gene assembly. Thus providing deeper interactions and a corresponding potential biological mechanism.^11^

In this study, we showed the trajectory of the gut microbiome with age and constructed a gut microbiome aging clock on an expanded large-scale metagenomic dataset. We defined the gut-aging clock as an age prediction model using gut microbiota as aging markers, whose age prediction values can serve as the biological age of individuals, achieving evaluation of aging status. We demonstrated that there is a universal association between gut microbiota and age and identified the key microbiota markers associated with it and the degree of influence. Biological age based on gut microbiota (gAge) prediction model was applied to an elderly population for aging assessment, and gAge and its prediction residuals could effectively represent the aging population. The results of this study may help improve the understanding of the relationship between microbes and age. Based on the association between gAge and frailty, it is expected to realize the early identification of unhealthy aging through noninvasive flora detection and combine with the gut microbiome and its metabolic characteristics to conduct targeted intervention and regulation. This study provides new insights into the mechanisms and targeted interventions of the gut microbiome during aging.

## Results

### Age-specific characteristics of gut microbiome taxa and metabolic pathways

To determine the potential gut microbial aging markers that are consistently altered with age, we performed a systematic analysis based on large-scale gut microbiome metagenomic datasets in a total of 55 study cohorts, with ages ranging from 18 to 107 years.

**Figure 1.**
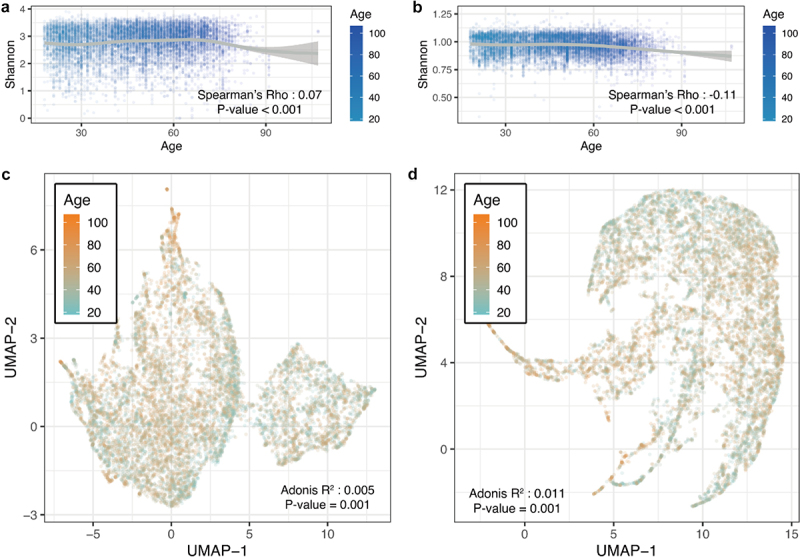
Gut microbial characteristics of different age groups. (A) Scatter plot of microbial species Shannon index for age. (B) Scatter plot of microbial pathways Shannon index for age. (C) UMAP plot of the microbial species using Bray–Curtis distance. (D) UMAP plot of the microbial pathways using Bray–Curtis distance.

Microbial diversity was measured to evaluate whether there were age-specific trends in microbiome composition and function. The α-diversity displayed a significant correlation (*P* < .001 for species and pathway) between the different age populations (Figure 1a, b). Species diversity increased with age (Spearman’s *P* < .001, both in richness and Shannon index), while metabolic pathways decreased (Spearman’s *P* < .001). Uniform Manifold Approximation and Projection (UMAP) was used to perform unsupervised clustering of bacterial beta diversity (Figure 1c, d). After adjustment for host-independent confounders, age exhibited a significant effect on the structure of the gut microbiome (Adonis *P* =.001). These results demonstrated that the taxa and functions of the microbiota change dynamically over time.

**Figure 2.**
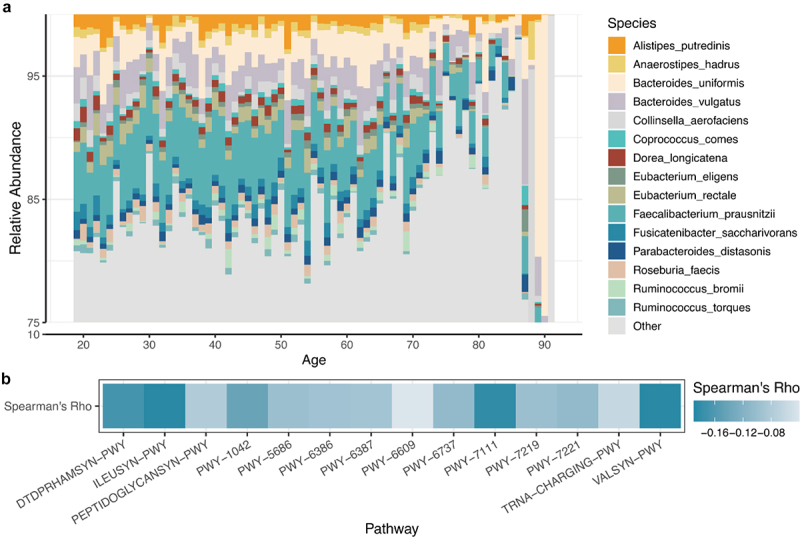
(A) barplot showing the relative abundance of the top 15 species for each age. (B) The correlation coefficient between the top 15 pathways and age.

The predominant microbiota components also showed a significant shift among the different age groups (Figure 2a). Most species exhibited significant correlations with age, and the relative abundances of *Faecalibacterium prausnitzii*, *Eubacterium rectale*, and *Ruminococcus torques* decreased with age. Pathways also exhibited significant age-related changes similar to species (Figure 2b); L-valine biosynthesis (VALSYN-PWY), L-isoleucine biosynthesis (ILEUSYN-PWY), and pyruvate fermentation (PWY-7111) were strongly negatively correlated with age. Our findings indicate that both the species components and metabolic pathways of the gut microbiota are correlated with the aging process.

### Aging clock based on an integration method can import the utilization of heterogeneous data while achieving higher accuracy

**Figure 3.**
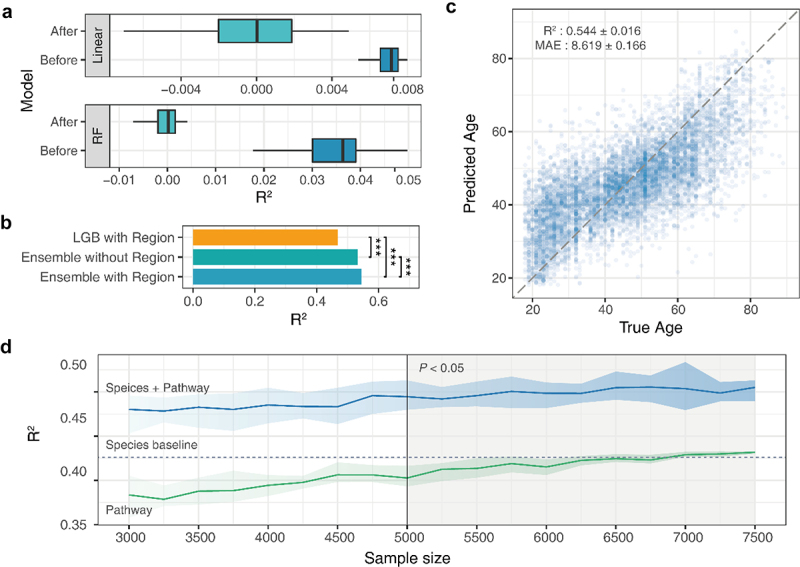
Performance evaluation of the ensemble model based on gut microbiome data (A) two heterogeneous models (linear and RF) were established to evaluate the c clustering and filtering. (B) Performance differences between integration strategies. (C) Ensemble model age prediction performance. (D) Impact of unpaired data on ensemble model performance.

Abbreviations: RF, random forest

Our previous study used a multi-view data-adapted ensemble machine learning framework. Through the integration of heterogeneous models and data, we observed broad patterns of gut microbiome changes during aging and clarified the impact of species and functions in this process.^12^ To reduce the influence of age-independent factors, a fine-grained stepwise methodology was adopted to decrease confounding effects on the microbiome profile (see Materials and Methods; Fig. S1). Subsequently, most of the samples were retained. Similarly, two heterogeneous models (Linear and Random forest, RF) were established to evaluate the correlation between the age and region of the corrected dataset (Figure 3a). After clustering and filtering, there was no obvious correlation between the regional label and the age of the retained sample (R^2^ of all models was less than 0.001).

Next, an ensemble prediction model developed in our previous study was used to characterize the biological age of the individual gut microbiota. We found that, compared with the single-factor prediction model, the multi-factor ensemble can significantly improve the prediction accuracy of the aging clock (Figure 3b). To verify the performance of the ensemble model, 5-fold cross-validation was employed, and the results demonstrated that the mean absolute error (MAE) was 8.619 years, and the average coefficient of determination (R^2^) was 0.544 (Figure 3c). These results indicate that the characteristics of the gut microbiome effectively predict chronological age in the population. Another interesting thing is, After comparing the predictive ability of modeling using all regional microbiome data (with regional label) and the same regional microbiome data subset (without label), we find that based on all region information shows higher prediction accuracy at the four regional levels of the six datasets, and only slight performance degradation is observed at the other two regional levels (Δ R^2^ <0.05) (Fig. S2); Especially, there is a significant performance improvement for regional data with small sample sizes.

The advantage of independent modeling is that it requires only completely paired data during the training process of the final generalization model. There is no requirement for datasets during the upfront modeling process. We simulated the sample mismatch situation based on metagenomic data of the gut microbiota. We continuously increased the sample size of the metabolic pathway data after a fixed number of species samples (3000 samples) to compare the changes in model performance under non-pairwise modeling. The results showed that when the number of mismatched metabolic pathway data increased to 5000 or more, the performance of the ensemble model was significantly higher than that of the original pairwise sample data (Figure 3d). Moreover, the results of single-pathway modeling proved that the improvement in performance was not caused by more single-pathway data. We found that with the increase in the metabolic pathway sample size, the prediction accuracy was still lower than that of species composition. This finding shows that an independent modeling strategy can effectively improve the utilization rate of unpaired gut microbiota data, thereby contributing to the comprehensive utilization of gut microbiota data, especially multi-omics data.

### Identification of age-specific taxonomic and functional gut microbiome markers

**Figure 4.**
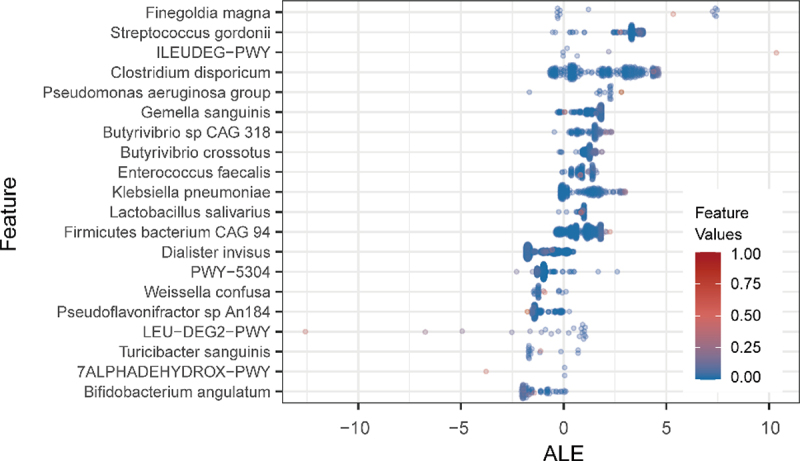
The top 20 age-specific markers. Beeswarm plot showed the distribution of accumulated local effect values under different marker abundance values.

To identify the key age-related gut microbiome characteristics of the ensemble model, the accumulated local effect (ALE) method was used, which is a model-agnostic interpretation method. It has a high degree of flexibility that can be used to analyze complex black box models. A series of gut microbiome characteristics that were significantly correlated with age was obtained (Figure 4). The markers identified in the large-scale dataset in this study were highly consistent with the results of our previous research. *Finegoldia magna* was still the highest effect factor for model-predicted age. In addition, *Pseudomonas aeruginosa*, *Enterococcus faecalis*, and *Lactobacillus salivarius* were also considered as potential key components affecting the gut aging clock among the two research results. However, the metabolic pathways of microbial communities also showed the same characteristic trends, especially for changes in the metabolic capacity of amino acids. The ability to metabolize branched-chain amino acids (BCAAs), such as L-leucine (LEU-DEG2-PWY) and L-isoleucine degradation (ILEUDEG-PWY), which are closely related to the health of the elderly, demonstrated a stronger influence on the aging clock, which is consistent with previous research.^13,14^ Compared with the commonly used feature interpretation metrics, such as the feature importance score and mean decrease accuracy, ALE allowed the evaluation of the directionality of the feature’s impact on the prediction of the result while ranking the importance of features.

### The gut aging clock reflects the health status and frailty of the host

**Figure 5.**
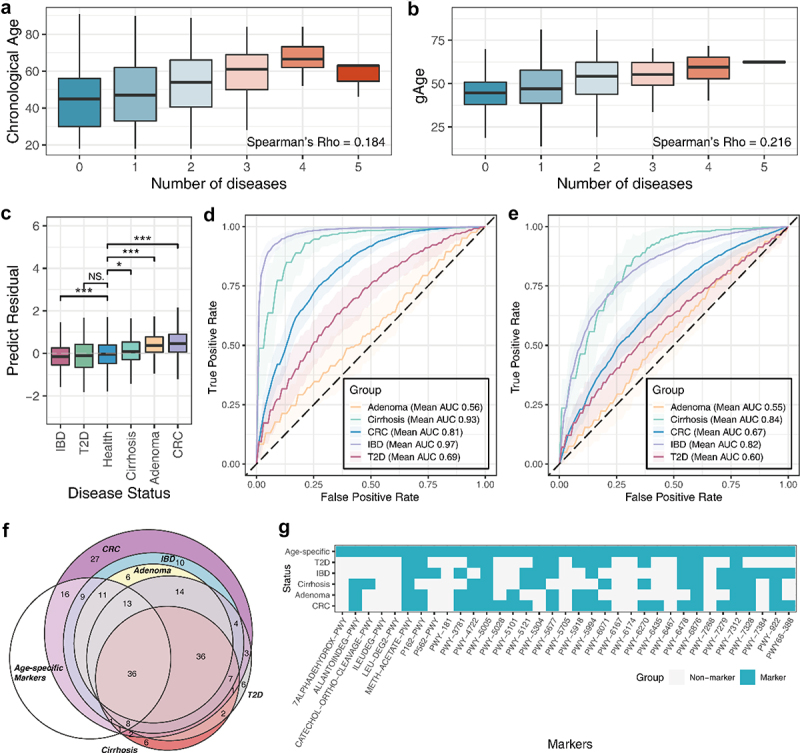
The gut aging clock reflected the health status of the host. (A) Box plot indicating the correlation between the number of diseases and chronological age. (B) Correlation between the number of diseases and predicted age. (C) Box plot indicating the significant differences in age-predicted residuals between disease states. NS: not significant, *P* >.05; *: *P* <.05; **: *P* <.01; ***: *P* <.001. The receiver operating characteristic (ROC) curves of the disease discrimination model constructed based on (D) species and (E) metabolic pathways. (F) Venn diagram indicated the degree of overlap between age-related and the diseases marker species. (G) Heatmap indicating the present condition of functional markers in the aging clock and diseases.

Abbreviations: IBD, Inflammatory bowel disease; T2D, Type 2 diabetes; CRC, Colorectal cancer.

To gain further insight into the relationship between dynamic changes in the gut microbiome and the host state, we first compared the correlation between the predicted age based on the aging clock and the number of host diseases (Figure 5a, b). The results of the regression analysis showed that the predicted age had a higher correlation with host multimorbidity than with chronological age (*P* < .001). Next, we examined the perturbation of disease states on the outcome of the aging clock (Figure 5c), and the results showed that, except for type 2 diabetes (T2D), other diseases will affect the predicted age, and most diseases will significantly increase the aging clock (*P* < .05).

Next, the markers explained by the aging clock were used to construct a discriminative model for each disease (Figure 5d, e). The results indicated that age-specific markers can effectively distinguish between diseased and healthy populations, suggesting that these markers are closely related to the host disease state, particularly the species characteristics that showed high accuracy in many diseases. A detailed comparison of the structure of the markers between each disease discriminant model and the aging clock exhibited a high degree of overlap (Figure 5f, g). Consistent with the results of previous analysis, there are significant differences in the abundance of related biomarkers in healthy populations and related disease populations (*P* < .05) (Fig. S3,S4).

The aging clock identifies almost half of the disease species markers and similarly, at the level of metabolic pathways. Notably, age-related pathway markers unique to the aging clock may reflect the potential association between the aging process and its metabolic capacity or metabolites, such as cholate (7ALPHADEHYDROX−PWY), L-leucine, and L-isoleucine degradation (LEU-DEG2-PWY, ILEUDEG-PWY).

### Gut aging clock and its residuals can reflect host aging stages

**Figure 6.**
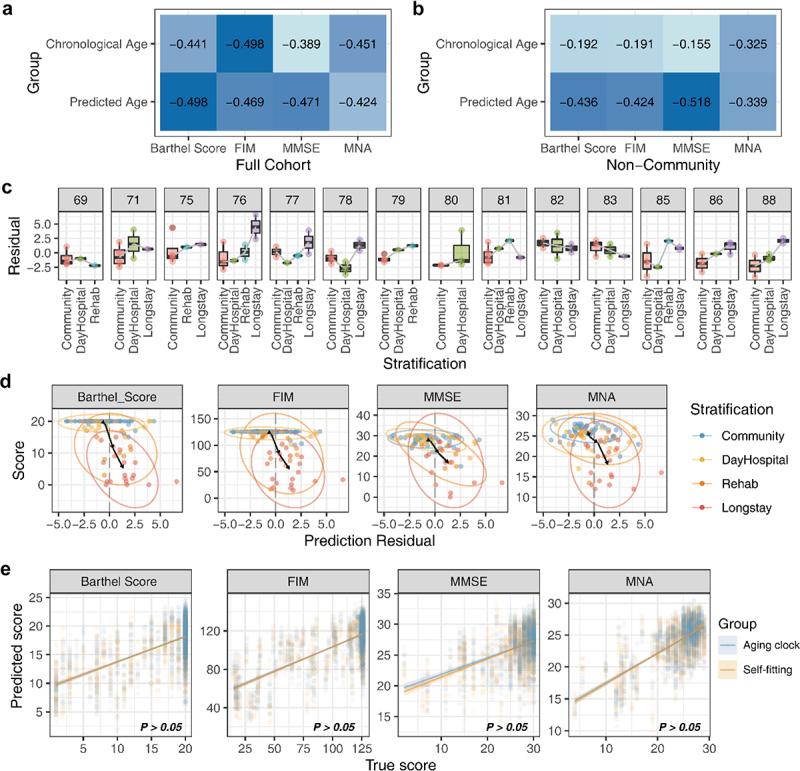
The gut aging clock reflected the frailty of the host. (A) Heatmap indicating the correlation of four frailty indices and two age types (functional independence measure, FIM; mini-mental state exam, MMSE; mini nutritional assessment, MNA). (B) The frailty correlation of the non-community population, including hospital days, rehabilitation (rehab), and long stay. (C) Box plot indicating the distribution trend of the prediction residuals and stratification in each age group. Only considered the age group with three or more different stratification categories. (D) The distribution characteristics of the prediction residuals and frailty scores of the cohort, and both indices obtained significantly high spearman’s correlation coefficient between the residuals and scores (*P* <.05). (E) True frailty score versus predicted score by various methods obtained microbiome markers. There was no significant difference between the prediction outcomes of the two source features (Wilcoxon *P* >.05).

To verify the connection between the aging clock and frailty in the elderly, we used an additional independent cohort for the study of the gut microbiome in the elderly population, which included a series of evaluation indicators for the body and cognition of the elderly.^15^ Considering the age distribution characteristics of the verification cohort, a targeted age-prediction model was built using data from the elderly population. The results showed that, compared to chronological age, the predicted age indicated a higher correlation with some frailty metrics (Figure 6a). A closer analysis demonstrated that for the aging population in the cohort, the predicted age exhibited a significant correlation with its frailty indices (Figure 6b). Afterward, the trend of prediction residuals in different frail stratifications was explored, and the results showed that in most age groups, older people with higher levels of care had greater residual deviations (Figure 6c). Furthermore, we investigated whether the aging-related indices and the prediction residuals indicated the same pattern in each stratification and found that in all measure metrics, people with a high level of care had greater positive prediction bias. The residual change trajectory of different groups of elders also pointed out that with the improvement of the level of care, the predicted age was higher than that of the community population (Figure 6d). Finally, to determine the generalizability of the gut aging clock, a comparison was made with a predictive model of aging indicators built using the validation cohort (Figure 6e). The results showed no significant difference (*P* > .05) in the prediction results between the aging clock and the validated cohort model.

Thus, the above results showed that the contribution of the aging clock predicted age to healthy states and frailty indices was significantly stronger than the chronological age, and this situation showed the potential relationship between the composition of the gut microbiome and the aging process for the host.

### The gut aging clock can be used to assess the degree of aging in an independent cohort of elderly population

**Figure 7.**
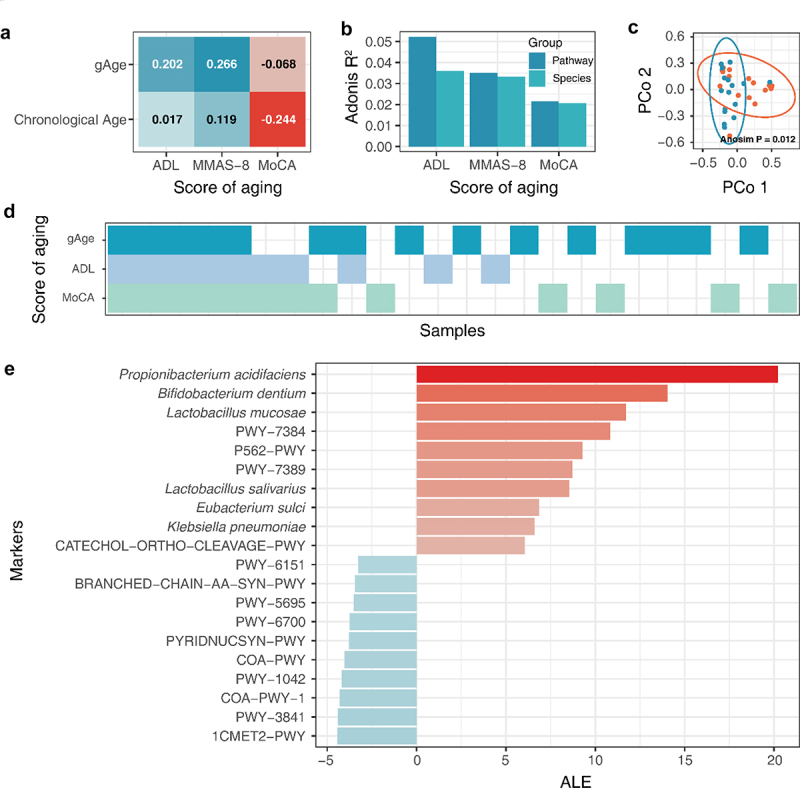
gAge assesses the degree of aging in the elderly population. Aging assessment based on gAge. (A) Correlation of different age characterization methods with aging scores. (B) Effect of different aging scores on gut microbiome. (C) Differences in species composition of different residual population. (D) Results of different aging evaluation methods. (E) Correlation between host background factors and indicators of frailty. (F) Aging marker identification based on cohort of elderly population.

We recruited a cohort of older adults to form an elderly cohort. The cohort was established at Wuxi People’s Hospital Affiliated to Nanjing Medical University, and was approved by the hospital ethics committee (approval number: KS2019039). A total of 32 elderly people were included in this experiment, and basic information on the cohort was obtained (Tab. S1). The study cohort included 18 males and 14 females. The average age was 69.03 ± 6.49 years old, the average height was 163.19 ± 7.81 cm, the average weight was 68.813 ± 11.69 kg, and the average BMI was 25.71 ± 3.17 kg/m^2^. We used scales to measure the indicators of aging in the aging population. The Activities of Daily Living Assessment Scale (ADL) was used to assess the level of disability in the elderly population.^16^ The Montreal Cognitive Assessment Scale (MoCA) was used to identify the level of knowledge barriers among the elderly.^17^ Morisky Medication Adherence Scale-8 (MMAS-8) was used to evaluate adherence to medication for disease treatment in the elderly^18^.The gut microbiota integration age ensemble machine learning model was used to evaluate gAge in the cohort population. Statistical analysis showed that compared with chronological age, gAge significantly improved the correlation between ADL and MMAS-8 (Figure 7a). Analysis of aging scores and differences in gut microbiota structure indicated that the MoCA score had the lowest degree of explanation for gut microbiota species and metabolic pathway structure, which may account for the low correlation between the gAge and MoCA scores (Figure 7b). The results of gAge prediction residual analysis of the elderly population showed that there were significant differences in the species composition of the gut microbiota among populations with different gAge residuals (*P* < .05; Figure 7c). Simultaneously, we found that serum alkaline phosphatase and insulin levels were significantly different in the elderly population with different gAge residuals. In addition, some studies have shown that serum alkaline phosphatase levels can predict the risk of cardiovascular and cerebrovascular diseases.^19^

As different aging evaluation indicators focus on different frailty phenotypes, the evaluation results of different indicators are different. The analysis of the aging population identified by ADL, MoCA, and gAge showed that the aging population identified by gAge overlapped with both ADL and MoCA scores, and the population that met both ADL and MoCA aging evaluations had a higher possibility of gAge bias (Figure 7d). This result suggests that individuals with a higher degree of aging may have a greater gAge positive bias. In general, gauge has been successfully used to evaluate the degree of aging in the elderly population.

### Analysis of marker species and pathways in the specific elderly population

To discover the characteristics of gut microbiota associated with aging in an elderly cohort, the ALE method was used to interpret the characteristics of the gut microbiota based on the data of the elderly population (Figure 7e). As ALE is a model-independent feature interpretation method, it can only interpret the model based on the given data. ALE can achieve targeted feature analysis of elderly population data and identify key species and metabolic pathways that affect gAge prediction results in specific populations. The results showed that *P. acidifaciens* and *Bifidobacterium dentium* etc. were key species associated with frailty in the elderly population. This result is consistent with the analysis of big data of the gut microbiota. *P. acidifaciens* and *Bifidobacterium* dentium have been identified as differential species associated with aging in the oral cavity of the elderly population.^20^

Anaerobic energy metabolism (PWY-7384, PWY-7389), inositol degradation (P562-PWY) and CATECHOL degradation (CATECHOL-ORTHO-CLEAVAGE-PWY) are important metabolic pathways determining gAge; Studies have shown that host aging and insufficient dietary folate can jointly induce folate metabolism disorders, leading to a higher risk of cancer, and folic acid supplementation can help to slow down the related adverse effects of aging.^21,22^ In addition, the coenzyme A synthesis (COA-PWY-1, COA-PWY), glycolysis (PWY-1042), and BCAAs (BRANCHED-CHAIN-AA-SYN-PWY) synthesis pathways were negatively correlated with aging.

**Figure 8.**
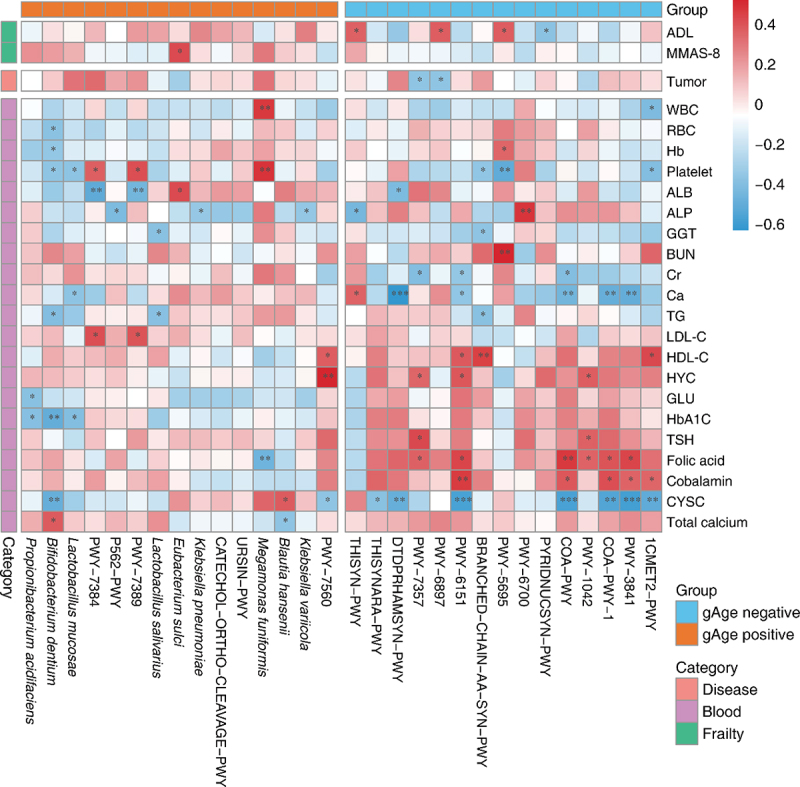
Analysis of correlation between markers of frailty and background factors in the elderly population.

The key species and metabolic pathways obtained from the interpretation of gut microbiota data in the elderly were analyzed using Spearman’s correlation with the host background factors, and the results showed that the identified key species were closely related to the degree of host aging, disease status, and multiple physiological indicators (Figure 8). Except for NAD synthesis (PYRIDNUCSYN-PWY), thiamine diphosphate synthesis (THISYN-PWY, PWY-6897), inosine acid degradation (PWY-5695)-related metabolic pathways, and ADL scores showed a significant positive correlation. The decrease in NAD levels contributes to the development of age-related diseases, such as metabolic and neurodegenerative diseases and cancer. However, increased NAD levels have been shown to prevent aging and related diseases.^23^ E. sulci was significantly associated with the MMAS-8 score, consistent with its effect on gAge, and was also an aging differential species in the mouth where its abundance increased with age.^24^ In terms of diseases, the thiamine phosphate (PWY-7357) and thiamine diphosphate synthesis pathways were significantly negatively correlated with tumors. Many studies have shown that subclinical thiamine deficiency occurs in patients with a variety of cancers.^25,26^

In addition, there were significant differences in the effects of gAge-related markers on the serum biochemical indices of the hosts. For example, gAge positively correlated markers showed higher positive correlations with hemoglobin, platelets, and LDL-C, whereas gAge negatively correlated markers showed higher negative correlations with creatinine, ionized calcium, and Cystatin C, and higher positive correlations with serum urea, HDL-C, folate, and vitamin B12. In conclusion, gAge-related gut microbiota species and metabolic pathway markers are closely related to host aging and may be potential therapeutic targets.

## Discussion

In this study, we obtained a reliable gut-aging clock based on large-scale metagenomic data, using an ensemble model. It only requires completely paired data during the training process of the final generalization model; there is no requirement for datasets during the upfront modeling process. This independent ensemble model can effectively improve the utilization of unpaired gut microbiota data. In addition, the predicted results of the aging clock reflected frailty. We identified 164 marker species and 35 marker pathways that were closely associated with host age changes and grouped them into accelerated aging or mitigated aging clusters according to the extent of their effect on the gut aging clock. Overall, we generated an aging clock that allowed us to further clarify age-related biomarkers and to discover that these species and pathways had important effects on host health and frailty states, the “gut age” obtained by the aging clock tracked a variety of aging phenotypes. Thus, this metric may serve as a potential diagnostic tool for identifying the health of a host, especially the risk of frailty.

We characterized the age-related markers, and ALE was applied for the interpretation of the ensemble model, which can have an impact on model prediction results under different feature value conditions, implying a shift from a qualitative assessment of ranking to a quantitative evaluation of the impact on prediction results. Another point to note is that two degradation pathways of BCAAs showed high importance but a distinct impact: isoleucine degradation demonstrated accelerated the aging clock with increasing abundance, whereas leucine degradation showed the opposite. It showed an accelerating effect in low-level abundance, but with an increase in abundance, it reversed and showed a mitigating effect. However, recent studies have demonstrated that BCAAs do not show complete beneficial effects; restriction of dietary BCAAs can delay aging and increase lifespan.^14^ This may explain why the highly-abundant leucine degradation pathway was beneficial in mitigating the aging clock. In general, these results suggest that the abundance levels of microbiome species or pathways must be maintained within an appropriate range because deviation from the proper dosage may have the opposite effect or adverse effects. The effect values based on the model interpretation may provide guidance for defining the extent of the intervention. In addition, we found that many marker species with a negative effect on the aging clock were rarely described in the human gut context and were instead common or isolated in the oral cavity, such as *Streptococcus gordonii*, *Propionibacterium acidifaciens* and *Streptococcus vestibularis*.^27–29^ This emphasizes the potential impact of the oral microbiome status on gut and host health.

We obtained a reliable model capable of accurately predicting host age based on large-scale gut metagenomic data. However, the model lacks evidence of a direct correlation with frailty, for which we first compared the association between the predicted outcomes and number of diseases (multimorbidity). Multimorbidity is a crucial indicator in evaluating the degree of accumulation of physiological damage during aging in an individual, and our results suggest that the predicted age was more responsive to multimorbidity than chronological age.^30^ Furthermore, an extra validation cohort stratified according to frailty and scale score was used, and the results demonstrated that the obtained predicted age reflects differences in the population across frailty states. Our findings sufficiently indicate that a model based on the gut microbiome can truly reflect the frailty of the host and can be used as a credible aging clock. In addition, we compared the overlap of signatures between the aging clock and each disease discriminant model, which may have potential aging intervention capabilities, such as cholate degradation. Previous studies have shown that the metabolites of bile acid are enriched by the gut microbiome in centenarians and can resist aging.^31^ Meanwhile, overlapping markers explained the response of the aging clock to the disease state. This means that the bias in the aging clock of the gut microbiome also represents the health status of the host, and higher predicted results may signal a unhealthy state, which explains the reason for the prediction bias in the non-aging population. We believe that this feature can help broaden the scope of applications for aging clocks.

The gAge prediction model was applied to the recruited elderly population for the aging evaluation. Through targeted interpretation of gut microbiota data in the elderly population, P. acidifaciens, Bifidobacterium dentium, Lactobacillus mucosae, Lactobacillus salivarius, Eubacterium sulci, and K. pneumoniae were found to be key species associated with aging in older populations. Anaerobic energy metabolism (PWY-7384, PWY-7389), inositol degradation (P562-PWY), CATECHOL degradation pathway (CATECHOL ORTHO-CLEAVAGE-PWY), and folate synthesis pathway (1CMET2-PWY, P562-PWY) PWY-3841) are important metabolic pathways associated with aging. In addition, the identified key species and metabolic pathways were also correlated with several physiological and biochemical indicators in the elderly. The results showed that gAge and its predictive residuals can effectively characterize the aging population.

Taken together, our results demonstrate the feasibility of the metagenome-based aging clock construct and the necessity for feature interpretation. gAge and its predicted residual can be used as a biological age indicator to cope with aging. Combined with the validation cohort, it was shown that gAge is closely related to multiple diseases and multiple population frailty indices. Our predictive analytics process improved our understanding of the differences and associations between the gut microbiome and the aging process. We expect our research to provide some reference or inspiration for targeted interventions in the aging process.

## Methods

### Taxonomic and metabolic pathways annotating of the metagenomic sequences

Our analysis was based on public metagenome data, which included a total of 56 study cohorts, of which 55 cohorts (including a multi-omics cohort) used for the primary analysis from a pre-process database,^32^ and 1 cohort served as the additional validation cohort with aging information.^15^ Specifically, validation samples were downloaded from the NCBI sequence data and annotated according to the following method.

To avoid interference of low-quality reads and adapters on annotation and subsequent analysis, Trimmomatic 0.39 was used to filter and retain high-quality data.^33^ The trimmed reads were then mapped to the human reference genome (hg38) using Bowtie2 2.4.4, samtools 1.12, and bedtools 2.30.0, to remove the host genes.^34–36^ The clean reads were applied to MetaPhlAn3 (mapping to the mpa_v30 database) and HUMAnN3 (mapping to the full UniRef 90 database)for taxonomic and functional annotation, respectively.^37^ The HUMAnN3 annotated metabolic pathways profile was further normalized to the relative abundance.

### Characterizing the changes of gut microbiome species and pathways with age

Composition analysis was performed using R 4.1.0. α-Diversity was calculated including Richness and Shannon index, and Spearman and Kruskal – Wallis tests were performed to evaluate the correlation and difference between α-diversity and age. The Python package umap-learn was used to conduct UMAP based on β-diversity.^38^ Permutational Multivariate Analysis (Adonis) was performed to evaluate the differences in microbial composition, and non-host-related factors were adjusted in Adonis as confounding factors (including DNA extraction kit and sequencing platform). The Kruskal – Wallis test was performed to evaluate the differences between the principal coordinates and age categories. Diversity was calculated using the R package vegan 2.5–7.^39^

### Effect of host covariates on the composition of the gut microbiome

Adonis was used to evaluate the degree of influence of each covariate on the gut microbiome, including species and metabolic pathways (covariates that existed in more than 50% of the samples were considered for analysis) and corrected by the aforementioned non-host-related factors.

### Data filtering for geographical factor

To filter geographical factors that were not related to age information, a previously published screening method based on country clustering and recursive elimination was used, and fine-tune optimization was carried out on this basis.^12^ As geographical factors and dietary habits were the main influencing covariates, other than age, the non-westernized population in the sample was first removed (only a low percentage of the total sample). Second, we clustered the country labels based on geographical location using the same method^12^ as before. Then, an optimized subset selection strategy was used, and compared with the method in the previous study, the recursive elimination of data sets improved from the subregion level to the single data source. This means that in each iteration, the feature eliminates the process using a single research cohort as the basic unit instead of cohorts with the same subregion label. Finally, heterogeneous models were used to evaluate the relationship between the selected subregion labels and age of the samples; R^2^ was less than 0.001 as the termination threshold for filtering.

### Machine learning construction and feature selection method

A set of heterogeneous machine regression models was constructed using Python, version 3.8.3. Linear regression (LR), Lasso, Ridge regression (ridge), Bayesian ridge regression (BR), elastic net (EN), k-nearest neighbors (kNN), linear support vector machine (LSVM), support vector machine (SVM), Decision Tree (DT), Random Forest (RF), and Gradient Boosted Regression Trees (GBRT) were constructed using the Python package scikit-learn 0.23.1.^40^ eXtreme Gradient Boosting (XGB and XGBRF) was built using the Python package xgboost 1.1.1.^41^ Light-gradient boosting was performed using the Python package lightgbm 3.0.0.^42^ Before constructing the machine learning models, all training data were standardized (Z-score). 10 times 5-fold cross-validation was used to evaluate the accuracy (R^2^ as the evaluation metric) of the model prediction.

The ensemble model was constructed according to a previously published method^12^, and LR was used as the weight-learning model.

Feature selection methods were implemented using Python. Univariate linear regression (FR), mutual information estimation (mutual), and embedding selection methods (RF, GB, XGB, and LGB) were based on the Python package scikit-learn. Pearson and Spearman’s correlation selection methods were implemented using the SciPy 1.5.0.^43^

### Ensemble model feature interpretation method

The accumulated local effect (ALE) method was used to explain the ensemble model. The ALE algorithm was implemented based on the modified Python package alibi 0.5.6,^44^ including warping the ALE function the package provides to make it compatible with multiple datasets and the parallelization of the feature interpretation process to improve speed.

In view of the results obtained from the interpretation, the average effect was higher than 0.083 (the effect value equals the magnitude of the effect on the predicted outcome, that is, the age deviation of the prediction, and 0.083 is equivalent to a prediction deviation of about one month made by the model). The absolute value of the filtering threshold of 0.083 indicates the deviation of the feature, and this feature was present in at least 1% of the sample, which was considered to be a potential age-specific marker.

### Additional cohort validation analysis

The Python package scipy was used to calculate the Spearman correlation between different age groups and frailty indices. The prediction residual was obtained by performing linear regression fitting on the predicted and chronological ages. In addition to building the gut microbiome aging clock, other model constructions, including classification and regression models, used the Python package LightGBM. The feature interpretation of LightGBM is based on the feature importance ranking of the model itself.

### Cohort recruitment and sampling of elderly population

The recruitment of subjects in the elderly population cohort was carried out through Wuxi People’s Hospital Affiliated to Nanjing Medical University, and was approved by the hospital ethics committee, and its ethical approval number is KS2019039.

The inclusion criteria were as follows: ability to understand and sign a written informed consent. Subjects were excluded from the study if they met any of the following criteria: severe infection, surgery, severe cardiovascular and cerebrovascular diseases, and severe trauma. Patients with acute intestinal diseases, combined with rheumatic and immune diseases, and other cognitive or mobility impairments due to serious medical conditions, have used antibiotics in the past three months or have participated in other clinical trials; do not consent to or are unable to perform the duties of a participant in accordance with the requirements of the study, including performing physical and blood biochemical tests, and providing complete information on past medical history.

Fecal samples were collected as follows: (1) the information of the subjects was marked on the body and cover of the stool sampling tube; (2) to avoid contamination of stool samples by environmental factors, samples were collected in clean toilets or other containers; (3) to avoid urine contamination of fecal samples, fecal samples were collected after urination; (4) after completion of sample collection, samples were stored in a refrigerator at −80°C within one hour; and (5) dry ice was used for cold storage during transportation, and the entire transportation process was protected from light and heat.

Blood samples of subjects were collected, and blood biochemical indexes were detected by automatic biochemical analyzer, including: White blood cell, red blood cell, hemoglobin, platelet, albumin, alkaline phosphatase, r – Glutamyltransferase, alanine aminotransferase, aspartate aminotransferase, urea, creatinine, ionized calcium, total calcium, triglycerides, low density lipoprotein cholesterol, high density lipoprotein cholesterol (HDL-C), homocysteine (Hcy), uric acid (UA), blood sugar, and serum islet.

### Metagenomic sequencing and analysis methods for specific population cohorts

A total of 32 fecal samples were tested by Shanghai Meiji Biomedical Technology Co., Ltd. Whole genome sequencing was performed using the Illumina NovaSeq 6000 platform. The acquired raw data were first subjected to quality control, and the processing process was as follows. First, the low-quality sequences were filtered using Trimmomatic 0.39 software. The average base mass in the sliding window was counted as 4 bp starting from the 5 ‘end of the sequence, and an average mass of 25 was used as the cutting threshold. Filtered sequences longer than 60 bp were retained as output for quality control.^33^ Second, the filtered sequences were aligned with the human reference genome (Homo sapiens genome assembly GRCh38, hg38) using Bowtie2 2.4.4, samtools 1.15 and bedtools 2.30.0 software. The host-derived genes present in the samples were removed.^34–36^ MetaPhlAn3 and HUMAnN3 were used for species and functional annotation of high-quality sequences after quality control,^45^ For HUMAnN3, the combined double-end data were used for annotation, and the counts obtained by annotation were normalized to the relative abundance results.

## Data Availability

This study incorporates data from previously published studies. The vast majority of sequence data comes from curatedMetagenomicData3 repository, the taxonomic and pathway profiles were downloaded for the analysis in this study. The Eldermet cohort was used as the validation cohort data in this study, the raw sequence data was available at the European Nucleotide Archive(ENA) via accession numbers PRJEB37017, and the metadata was available as part of the original publication. *1. Adjusting for age improves identification of gut microbiome alterations in multiple diseases. All relevant codes (or scripts) used for this analysis are available at https://github.com/hcwang-jn/gut-age.
